# Using acousmatic storytelling to facilitate communication and social interactions in people living with dementia: An iterative exploratory 12‐week study

**DOI:** 10.1111/ajag.70052

**Published:** 2025-06-13

**Authors:** Soon Yi Chua, Justin Christensen, Renee Timmers, Julia Schauerman, Kathryn Rawling

**Affiliations:** ^1^ Department of Music & Healthy Lifespan Institute University of Sheffield Sheffield UK; ^2^ Creative Research in Sound Arts Practice University of the Arts London London UK; ^3^ Sheffcare Ltd Sheffield UK

**Keywords:** communication, dementia, memory, music, social interaction, storytelling

## Abstract

**Objective:**

Communication challenges in people living with dementia can limit social interactions with fellow residents and hinder professional carers' ability to provide person‐centred care. While research highlights the benefits of reminiscence, storytelling and music interventions in facilitating social interaction, the potential of using soundscapes in storytelling remains underexplored. This qualitative study investigated how acousmatic storytelling—using sound recordings and music to inspire storytelling, evoke imagery and emotion, and iteratively develop hybrid audio compositions—can enhance communication and social interactions in people living with dementia.

**Methods:**

Twelve one‐hour workshops were conducted with individuals in the early to mid‐stages of dementia at a care home, using auditory cues (music and soundscapes) and visual prompts (photographs and poems) to stimulate memory recall, storytelling and group discussion. Data were collected through video and audio recordings, participant observation and semi‐structured interviews with care workers.

**Results:**

Inductive thematic analysis identified two key themes in verbal communication: (1) reminiscence and (2) creative sound associations, as well as five themes for non‐verbal behaviours: (1) supporting verbal communication, (2) substituting verbal communication, (3) expressing emotions, (4) enhancing rapport with others and (5) expressing musicality. These themes highlight how participants engaged meaningfully in both storytelling and group interactions.

**Conclusions:**

Findings suggest that acousmatic storytelling offers a platform for individuals living with dementia to engage in verbal communication, employ purposeful non‐verbal behaviours and participate in meaningful social interaction. This highlights its potential as a narrative approach for enhancing person‐centred care in long‐term care settings.

**Practice impact**

This study highlights the potential of acousmatic storytelling as an innovative, relational approach to enhancing communication, social engagement and emotional well‐being among people living with dementia. By integrating music, soundscapes and storytelling, the approach offers practical guidance for care settings, supporting personalised care strategies and fostering meaningful interpersonal connections.

## 1 INTRODUCTION

Communication plays a critical role in promoting positive outcomes in care, particularly for individuals living with dementia, who may experience verbal communication challenges. Despite these difficulties, people living with dementia still express a strong desire for social connection and maintaining relationships.^1^ Many retain a strong capacity for non‐verbal communication, including facial expressions, gestures and non‐speech vocalisations.^2^ Reduced opportunities for meaningful interaction increase the risk of social isolation and may negatively impact the well‐being of individuals living with dementia and their quality of care, leading to unmet needs, increased frustration and distress for both the individual and their carers.^3^ This study explored ways to support communication and foster social interaction among care home residents living with dementia using artistic means, specifically acousmatic storytelling.

‘Narrative care’ highlights the role of storytelling in preserving and expressing personhood.^4^ It encourages people living with dementia to share their stories and experiences, promoting a deeper understanding between carers and the individuals they support. Acousmatic storytelling, originally developed by Amelidis,^5^ is the setting of recorded spoken word within composed sound scenes that can evoke particular emotions, time periods and/or locations. Schauerman has reimagined this genre as a participatory activity, exploring how recorded soundscapes and music can stimulate storytelling and evoke emotionally meaningful imagined scenes.^6^ This approach supports reminiscence and mutual understanding by enabling participants to revisit their own memories and connect with each other's experiences through sound.

Given the importance of communication for maintaining personhood, fostering connections and ensuring quality care,^7^ this study explored the use of autobiographical stories in a group setting. Specifically, it examined how reminiscence storytelling, enriched with music, soundscapes, poems and photographs as memory retrieval cues, can facilitate both verbal and non‐verbal communication and promote social interactions.

### 1.1 Storytelling and social engagement

Storytelling can be a powerful tool for stimulating autobiographical memory and fostering self‐expression, thus supporting meaningful communication and social interactions.^8^ In collaborative and supportive environments, storytelling enables individuals living with dementia to share life experiences, interests and feelings, enhancing their engagement and verbal communication.^9^ It can also promote social exchanges, including spontaneous comments, humour and responses such as singing songs that are meaningful to the group.^10^

Holm et al.^11^ demonstrated that people living with dementia not only shared their past experiences but also expressed curiosity and interest in others' stories, which fostered a sense of community among a group of six individuals living with moderate to advanced dementia and three paid carers in a home care centre setting. Initially, interactions occurred primarily between the program leader and the participants, but as sessions progressed, participants began to communicate directly with each other, highlighting the potential of storytelling to build connections.

Despite its benefits, some researchers argue that storytelling may not be suitable for individuals with severe communication difficulties due to cognitive and linguistic impairments.^12^ Hydén^13^ counters this claim, suggesting that even those with significant communication challenges can participate meaningfully in storytelling through non‐verbal means, and drawing on these resources can show individuals to be more agentive and competent than they may at first appear. Storytelling as an embodied experience allows individuals to use gestures and other physical cues to convey stories.^14^ These gestures, being used as substitutes for verbal narration, enable carers and listeners to effectively interpret and engage with the stories.^15^

### 1.2 Use of memory retrieval cues

Based on a review of relevant literature, a variety of sensory cues, such as visual, olfactory and auditory stimuli, have been shown to stimulate memory recall in reminiscence storytelling.^16^ Lopis et al.^17^ suggested that visual and olfactory cues are particularly effective to help individuals recall memories vividly, while Willander et al.^18^ highlighted the efficacy of visual and auditory cues. Although findings differ regarding which sensory modalities are most effective, music and soundscapes appear promising in their ability to evoke strong emotions and memories.^19^

### 1.3 The role of music in communication and social interactions

Music has been used to improve communication and facilitate social interactions in dementia care,^20^ increasing engagement both during and outside music sessions.^21^, ^22^ This may take the form of encouraging music‐evoked autobiographical memories (MEAMs), which stimulate conversations and expressions of emotions.^23^ Unlike deliberate recall, music‐evoked autobiographical memories often occur spontaneously, offering an avenue for individuals with dementia to engage despite cognitive challenges.^24^ Personalised music can provide opportunities for social contact, sharing of memories and stimulated conversations while also encouraging non‐verbal communication through clapping, tapping and facial expressions.

Beyond reminiscence, group music‐making activities are valuable for social engagement. Many individuals living with dementia may retain musical skills. Singing familiar songs and remembering musical details allow them to connect and express emotions even when verbal communication is challenging.^25^ Singing, in particular, helps regulate anxiety and promote dialogue, creating a positive state for interaction.^26^ This study examines how combining these effects can enhance social engagement among people living with dementia.

### 1.4 Current study

This study explored the potential of acousmatic storytelling, which integrates music, soundscapes and storytelling, to facilitate verbal and non‐verbal communication in individuals living with dementia. By using these cues, participants were encouraged to share and discuss their own stories and engage with others' narratives. The sessions were conducted in a residential care home in the north of England that accommodates up to 54 residents. The home includes communal lounges that provide a suitable environment for creative, group‐based activities such as the one explored in this study.

This exploratory study addressed the following research questions:

1. How do residents living with dementia engage in verbal and non‐verbal communication during acousmatic storytelling sessions, particularly when reminiscing and making creative associations to sounds?
2. What are the social dynamics and emotional responses among participants during the acousmatic storytelling sessions, and how do they develop over the 12‐week program?

## 2 METHODS

### 2.1 Study Design

This exploratory qualitative study examined how verbal and non‐verbal communication and social interactions develop during acousmatic storytelling workshops for individuals living with dementia. A qualitative approach was chosen as it allows for in‐depth exploration of participants' experiences and the creation of rich narratives that align with the study's objectives.^27^

### 2.2 Participants

The study involved 14 older adults (ages 72–94, mean age = 85.2) with early‐ to mid‐stage dementia, residing in a Sheffcare care home in Sheffield. Weekly session attendance ranged from 10 to 12 residents (with most residents attending between 8 and 12 sessions). Two participants withdrew from the sessions due to ill health, and one left after moving out of the care home. Additionally, four new residents joined after the workshops had started.

Participants were selected using purposive sampling conducted by care home activity workers based on residents' interest in music‐based activities. Alongside the residents, two care home activity workers, three student volunteers and four researchers (three female, one male) participated. Activity workers were chosen for their familiarity with the residents and their experience leading art‐based activities, facilitating a supportive and engaging workshop environment.

### 2.3 Materials

The workshops incorporated a range of auditory and visual prompts, including music from the 1950s to 1980s, soundscapes, poems and photographs. These cues were used to stimulate memory recall and generate stories and discussions. Initial sessions focused on music and soundscapes, with poems and photographs added later based on participant feedback. Materials were tailored weekly to reflect themes emerging from prior sessions, with the aim of enhancing engagement and relevance.

### 2.4 Procedure

The 12‐week program consisted of 1‐h acousmatic storytelling workshops led by Schauerman, a postgraduate researcher and composer trained in this method, with support by Chua, Christensen and Rawling. These sessions were also earlier piloted in 2021 by Schauerman and Rawling, with four other residents. Workshops followed a structured format and were iteratively developed based on participant feedback. Sessions began with a welcoming song, personalised greetings and an introduction to the day's theme. This was followed by ‘Sharing Stories’, which used prompts to spark discussions, and ‘Creative Responses’, which encouraged participants to create or choose sounds, songs or poems in response to the shared stories. Sessions concluded with a closing song. Themes explored included home, personal histories, vacations and past challenges. Participants were informed that the researchers were interested in exploring whether sharing sounds and making music together during the sessions could make it easier for them to communicate with each other. Prior to starting, information sheets and consent forms were explained to participants and shared with their families. Informed consent was obtained both from participants and their families. Participation was voluntary, with anonymity assured through the use of pseudonyms. Ethics approval was obtained from The University of Sheffield Department of Music (approval number 050790).

### 2.5 Data collection

Multiple methods were employed to facilitate comprehensive data capture. All workshops (60 min each) were video‐ and audio‐recorded using Insta360 ONE X2, Zoom Q4 and Zoom H2n/H1 recorders. The Insta360 provided continuous 360‐degree coverage for detailed interaction analysis. The first author conducted participant observations in six workshops, interacting directly with participants to understand communicative dynamics.^28^ Semi‐structured, audio‐recorded interviews were conducted upon completion of the sessions with care workers (30 min) and the workshop leader (70 min) (see Appendix S1 for interview questions).

### 2.6 Data analysis

#### 2.6.1 Verbal communication

Audio recordings from Workshops 3, 6, 9 and 12 were transcribed verbatim. Conversation analysis identified interaction patterns, strategies for managing conversations and outcomes.^29^ Inductive thematic analysis explored emergent meanings using Braun and Clarke's six‐phase method^30^: data familiarisation, code generation, searching for themes, reviewing themes, defining themes and producing the report.^31^, ^32^

#### 2.6.2 Non‐verbal communication

Video footage of key workshop segments was reviewed to capture eye gaze, facial expressions, gestures, posture and vocal tone. Musical gestures such as clapping and tapping were interpreted through the lens of Krøier et al.'s five interconnected themes—vitality, disciplined subjectivity, attunement, therapeutic presence and validation—with particular emphasis on attunement and validation.^33^ Behaviours were classified as sociable/neutral (promoting engagement) or unsociable (diminishing engagement).^34^ Thematic analysis was again applied.

### 2.7 Reflexivity

Chua transcribed the recordings, independently coded the themes and wrote up the study as her master's thesis under the supervision of Christensen and Timmers, a postdoctoral researcher and professor, respectively. All three researchers have training in qualitative research methods. Chua maintained a reflexive journal, documenting her thoughts, preconceptions and research process to enhance transparency and trustworthiness.^30^

## 3 RESULTS

### 3.1 Verbal communication during acousmatic storytelling

**FIGURE 1:**
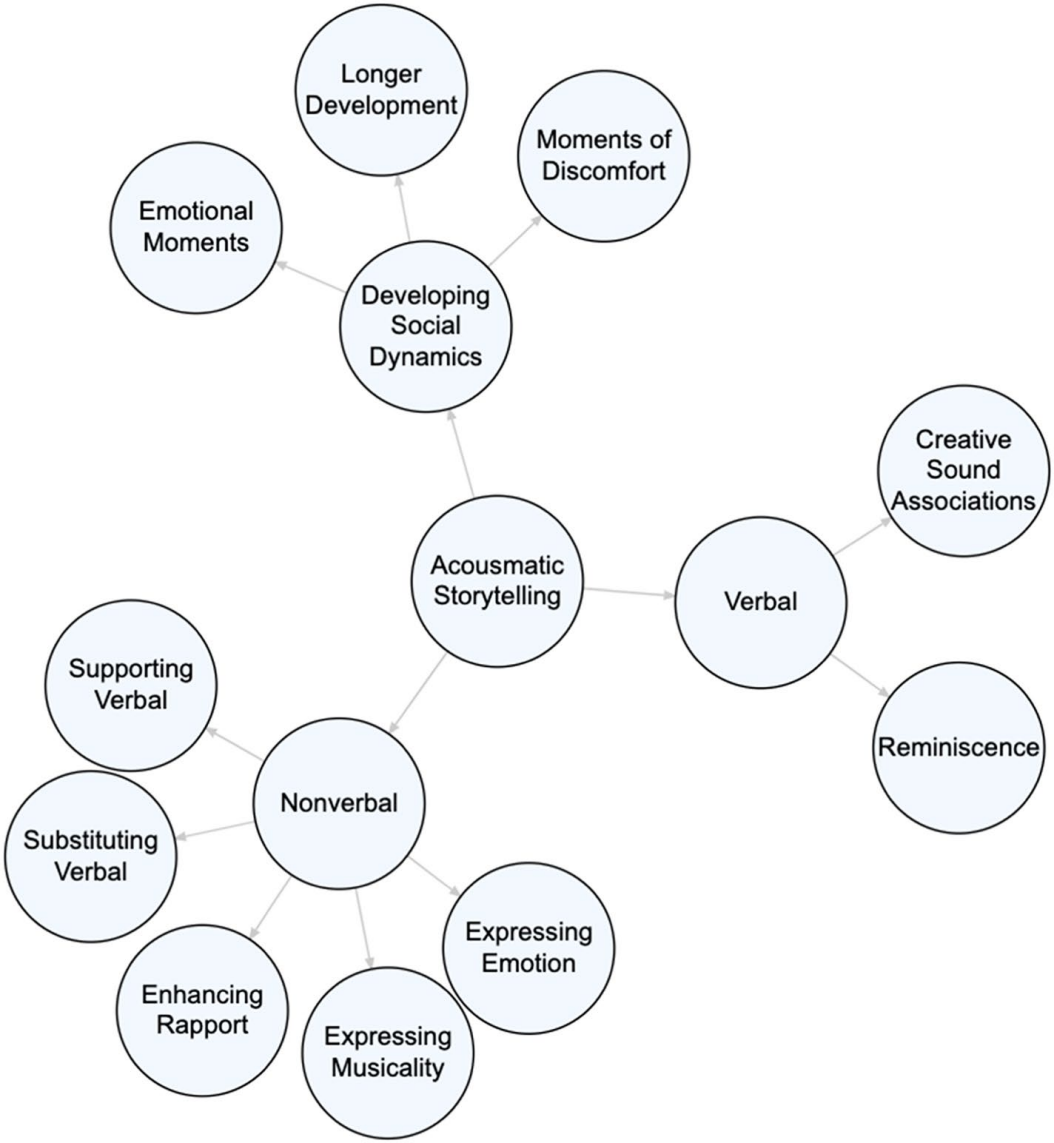
Themes and subthemes present in analysis.

As seen in Figure 1, thematic analysis of the transcripts revealed two primary themes in participants' verbal communication: (1) reminiscence and (2) creative sound associations.

#### 3.1.1 Reminiscence

Participants frequently reminisced about past experiences, either independently or with the facilitation of the workshop leader or care workers. For instance, Bethany shared detailed reflections on her holidays, providing evaluations and interpretations of her experiences in addition to describing the activities and emotions associated with them (Table 1, Quote 1).

**TABLE 1:** Quotes from participants from sessions.

Number	Quote
1	**Bethany:** Holidays abroad weren't for us at first until you'd saved hard and then you'd go with a group of people you know. And I liked it because I like the sunshine and I like lying in the sun, and I'm very lazy, and I like being waited on, which is lovely when you're going abroad. And they'll all come and say, ‘Yes ma'am, what do you want to drink?’ and it's a nice atmosphere. I think it doesn't matter where you go as long as you go and it's your holiday. As soon as you say it's holiday, you begin to enjoy it, don't you? Well, I do
2	**Care worker 1 (CW1):** Seaside places, what do you like? **Sandy:** Blackpool, Cleethorpes. You name it **CW1:** What were the best thing about them places? **Sandy:** When we went into the water and they shout ‘ah’ **CW1:** Did you go in and rescue them? **Sandy:** Yeah **CW1:** You said you like swimming in the sea **Sandy:** Yeah **CW1:** You must be quite a strong swimmer then? **Sandy:** I was, yeah. I would still do it now **Workshop leader:** What sort of swimming did you do? The crawl or the breaststroke? **Sandy:** Crawl. I'd still do it
3	**Workshop leader (WL):** Okay, Sandy, would you like to read that? What's on your card? **Sandy:** Yes. Felix, playing football, and dancing with him **WL:** Do you want to say anything else about that? Do you want to say who Felix is? **Sandy:** Felix is but no more… (Voice quivered) **WL:** And he was your…? **Sandy:** Husband. (Voice quivered) **WL:** Lovely. And you said he was a lovely man, didn't you? **Sandy:** Yeah **WL:** Okay, thanks for sharing. Do you want to say anything else about Felix? **Sandy:** Uh… No, I don't… Don't… like… doing it. No. (Sounded hesitant)
4	**Bethany:** How many brothers and sisters have you got? **Jody:** It's [the question is] private **Jody: [to care worker]** If she was a friend, you'd be able to ask her what she wanted [by asking this question]

In contrast, Sandy required more scaffolding, with the care worker and workshop leader posing a series of close‐ended questions to help her reconstruct her seaside holiday narrative (Table 1, Quote 2). While Sandy's narrative remains less detailed, it demonstrates the value of guided support in enabling participants to contribute to the storytelling process.

Occasionally, participants exhibited false beliefs or struggled to recall specific details during reminiscence. Kevin mentioned that he ‘still goes around decorating’ despite residing in the care home. Gretchen, on recalling her favourite music, said, ‘Not really, I've forgotten now. It's years ago’. These moments were met with patience and respect from the workshop leader and the group.

#### 3.1.2 Creative sound associations

The use of poems, song lyrics and photographs inspired participants to imagine potential corresponding sounds to these stimuli. In one session, participants came up with possible sound associations with the poem *The Tyger*, generating responses like ‘soft padding noises’ and ‘rustling’. In another session, participants made sound associations with the lyrics of *Annie's Song*, offering suggestions such as ‘waterfall’, ‘a lot of wind’, ‘little donkey’, ‘baby animals’ and ‘a yeti’ to evoke the lyrics ‘Like the mountains in springtime’. These exercises not only stimulated their imagination and verbal communication but also involved non‐verbal elements, such as gesturing to emphasise descriptions.

### 3.2 Non‐verbal communication during acousmatic storytelling

Participants demonstrated a variety of non‐verbal communicative behaviours during the sessions that either complemented, substituted for or enhanced their verbal interactions. These behaviours were categorised into five themes—Supporting Verbal Communication, Substituting Verbal Communication, Enhancing Rapport, Expressing Musicality and Expressing Emotion—as shown in Figure 1. The breadth and richness of participants' non‐verbal communication substantially contributed to their ongoing involvement and the positive atmosphere of the sessions. Detailed examples illustrating these communicative behaviours are provided in Table 2.

**TABLE 2:** Non‐verbal communication observed during acousmatic storytelling sessions.

Category	Examples of participant behaviours observed during sessions
Supporting verbal communication	Participants used gestures, facial expressions, and body movements to reinforce their speech. Nodding and head shaking frequently emphasised ‘yes’ or ‘no’ responses. Kevin supported his verbal question to Sandy about whether she ever had a pet by using a palm‐stroking gesture
Substituting verbal communication	When speech was challenging, non‐verbal gestures substituted effectively. For instance, Belle, having limited language production ability, made a tapping gesture with a soft toy to symbolise her presence when the workshop leader greeted her in the welcoming song. Participants often used gentle touch to gain another's attention in the group
Expressing emotions	Smiling and laughter frequently occurred during joyful interactions. Emily smiled widely after receiving a compliment on her poem reading. Gestures like the thumbs‐up were used by participants to signal approval and validation. Elsa, in particular, used this gesture throughout the sessions to convey her approval and agreement, reinforcing her connection with the group
Enhancing rapport	Participants regularly used body language and positioning to signal engagement and connection, including nodding, making direct eye contact, and physically turning their bodies toward others during conversations
Expressing musicality	Participants expressed musical engagement through gestures and spontaneous singing. Kevin made expressive gestures corresponding directly to lyrics when singing *Fly Me to the Moon*, including swaying his wrist to the beat, pointing upward at ‘Let me play among the stars’, extending his hand at ‘Hold my hand’, and pointing to himself at ‘Baby, kiss me’. Malcolm spontaneously sang *Morning Has Broken* when receiving the lyrics. Belle engaged non‐verbally by rhythmically tapping her hand to music, assisted by a care worker

### 3.3 Social dynamics and emotional responses

Participants' social interactions and emotional responses evolved over the 12‐week program, developing as they became more comfortable with one another and the storytelling format. Participants frequently built on each other's stories, contributing their own memories and experiences. For example, when Elsa shared her seaside memories, others chimed in with foods they associated with the beach, such as ‘cockles and mussels’ and ‘ice cream’, fostering a sense of shared experience. Additionally, participants developed their own unique ways of utilising non‐verbal behaviours, inventing and using alternative communicative resources.

#### 3.3.1 Emotional responses and moments of discomfort

Participants exhibited a range of emotional responses, from moments of joy to discomfort. Humour played an important role in fostering a positive atmosphere, as seen when Sandy jokingly addressed Kevin as ‘sir’, prompting his playful retort, ‘I am not knighted yet’. Sensitive memories sometimes triggered distress, such as Sandy becoming visibly upset when discussing her late husband (Table 1, Quote 3). Jody expressed discomfort with a task that involved asking personal questions, demonstrating her awareness of boundaries regarding privacy and highlighting the emotional complexity of the sessions (Table 1, Quote 4).

#### 3.3.2 Social dynamic development over the 12 weeks (as seen from interviews with staff)

Rawling, who has in‐depth knowledge of the residents' experiences both before and after the sessions, suggested that this work gave the residents their voice back and provided them with a new medium to share their life history. It helped residents reclaim a sense of importance while allowing them to enjoy the interest shown by others. The inclusive nature of this work encouraged the growth and flourishing of creativity over the 12‐week period, and it also greatly benefited the activity workers by adding to their skills.

The activity workers who participated in the sessions described them as having significantly contributed to the well‐being and social interactions of the residents involved. For example, they observed that the sessions increased residents' recognition of one another in the hallways. The practice of residents introducing themselves at the beginning of each session may have facilitated this by providing them with an opportunity to feel proud of their contributions and alleviating anxiety about the activities ahead, thereby fostering a more positive group atmosphere at the sessions.

Previously reserved residents demonstrated greater involvement over time. For instance, one individual who struggled to express emotions became increasingly engaged in group activities and was able to remain attentive for the entire duration of the sessions. Others transitioned from initial resistance or disengagement to active participation, sharing stories as their comfort with the sessions grew. The sessions also helped to moderate overly talkative residents, resulting in a more balanced group dynamic. Additionally, residents with hearing loss benefited from the use of small breakout groups designed to accommodate their needs, and staff noted that activities such as listening to nature sounds promoted focus and encouraged meaningful engagement with the material.

## 4 DISCUSSION

This study explored how acousmatic storytelling can facilitate verbal and non‐verbal communication and social interactions among care home residents living with dementia and how these interactions develop during and across the 12‐week series of workshops.

Three primary findings emerged: First, acousmatic storytelling provided a platform for residents living with dementia to reminisce and make creative sound associations where they could practice communication and relational skills. Second, most participants used non‐verbal behaviours purposefully to communicate with others. Finally, storytelling sessions cultivated a positive social atmosphere, where shared experiences fostered deeper connections and emotional expression among participants (even with the potential for discomfort), indicating the potential for acousmatic storytelling to be incorporated as a narrative approach to support social cohesion in dementia care settings.

### 4.1 Verbal communication

Participants' reminiscence during storytelling aligns with findings that remembering life events promotes communication among people living with dementia, as these memories are often retained longer than recent ones.^35^ Consistent with Fels and Astell,^36^ this study found that sharing significant life events provided opportunities for self‐expression and identity reinforcement. While some participants narrated elaborate stories independently, others required scaffolding from the facilitator and carers to articulate experiences. This echoes the findings of Mills^37^ and Usita et al.,^38^ who found that people living with dementia often benefit from interactional prompts to produce coherent narratives. Future interventions might consider training facilitators to use open‐ended questions, as suggested by Ihara et al.,^39^ to further encourage residents' storytelling autonomy.

A novel aspect of this study involved engaging participants in creative sound associations through poetic and musical prompts. Participants responded to sensory prompts with imaginative connections, reflecting prior findings that creative engagement fosters cognitive stimulation and enjoyment, especially when traditional reminiscence is challenging.^40^ Creative prompts may thus provide an alternative form of engagement for individuals with more advanced memory impairments.

### 4.2 Non‐verbal communication

Participants employed gestures, facial expressions and body language to support or replace verbal communication, highlighting the functional adaptability of non‐verbal communication among individuals living with dementia.^41^ Non‐verbal behaviours served to initiate, maintain and enhance conversations, consistent with previous findings.^42^ Participants demonstrated unique, personalised non‐verbal cues, including inventive prop usage to communicate, supporting the findings of Sabat and Harré.^43^ Conversely, some participants struggled to use non‐verbal cues effectively, suggesting a need for communication partners to adopt empathetic and observant approaches when interacting with such individuals.^44^ Music and singing appeared to facilitate expressive non‐verbal interactions, as participants synchronised their movements with one another, tapping and making gestures to familiar songs, resonating with Malloch and Trevarthen's^45^ concept of ‘communicative musicality’.

### 4.3 Social dynamics and emotional responses

Acousmatic storytelling fostered a supportive social environment where participants displayed a wide range of social and emotional behaviours. Participants developed rapport through turn‐taking, attempts to repair conversations, contributing to each other's narratives, validating each other's experiences with encouraging responses and using humour, supporting findings on shared narratives fostering social connection.^46^ The sessions also allowed participants to express diverse emotions, including joy, pride and occasionally discomfort when discussing sensitive topics like loss. The group setting allowed participants to both express and receive social validation, essential for promoting well‐being and dignity.^47^

### 4.4 Implications

Integrating acousmatic storytelling into care routines can foster social connections and communication. Becoming familiar with residents' unique communicative styles and non‐verbal cues can inform tailored care approaches, enhancing responsiveness to each resident's needs.^48^ Moreover, involving care workers in acousmatic storytelling sessions may improve relational caregiving by deepening their understanding of the past lives and identities of residents.

### 4.5 Strengths and limitations

A key strength of this study was its innovative use of acousmatic storytelling, enabling residents to communicate both verbally and non‐verbally. The facilitator's skill at an empathetic approach likely contributed to the sessions' success.^49^ However, reliance on auditory prompts limited engagement opportunities for participants with hearing impairments. Future studies could explore using personalised audio devices or adjustable sound levels to accommodate diverse needs.^50^ While conducted in a single care home, this study offers transferable insights into how sound‐based storytelling may support communication and connection in dementia care settings.

### 4.6 Future directions

Future research could adopt a longitudinal design to assess the lasting impact of acousmatic storytellings on communication and social engagement. Pre‐ and postintervention measures could evaluate the effectiveness of storytelling on verbal fluency, social participation and emotional well‐being. Additionally, exploring creative sound associations as a standalone intervention might provide alternative engagement methods for residents with advanced cognitive impairment.

## 5 CONCLUSIONS

This study highlighted the potential for acousmatic storytelling to facilitate verbal and non‐verbal communication, foster social interactions and support emotional expression in residents living with dementia. Narrative‐based innovative methods such as acousmatic storytelling in care facilities can enhance the quality of life for residents, supporting their personhood and social connectedness. While some outcomes may reflect the novelty of the activity and the intergenerational aspects of the visiting team, care home staff responded with enthusiasm and began incorporating elements of the approach into their ongoing work. With appropriate support for technology and facilitation, acousmatic storytelling holds promise as a sustainable and adaptable method within everyday dementia care practice.

## FUNDING INFORMATION

This research is funded by a UKRI Future Leaders Fellowship to project PI Jennifer MacRitchie (Grant Number MR/T040580/1), HEIF funding (Grant Number X/015090‐25), and a Transforming and Activating Places Studentship.

## CONFLICT OF INTEREST STATEMENT

No conflicts of interest declared.

## Supporting information


Appendix S1

## Data Availability

The data that support the findings of this study are available on request from the corresponding author. The data are not publicly available due to privacy or ethical restrictions.
