# Supplementary material for: Using acousmatic storytelling to facilitate communication and social interactions in people living with dementia: An iterative exploratory 12‐week study
Source: Australas J Ageing. 2025 Jun 13;44(2):e70052. doi: 10.1111/ajag.70052 (PMC12163556; doi:10.1111/ajag.70052)
Supplement: Supplementary file 1 — Appendix S1 [file AJAG-44-0-s001.docx]

**Appendix A**

**Open-ended qualitative interview questions**

**Questions for care home workers involved in the sessions**

1. Which of the activities do you think were successful in connecting with the participants?
2. Which did not, and why do you think that was?
3. What do you think you would need in terms of guidance and resources to continue running the sessions?
4. Can you describe the residents' verbal and nonverbal communication before the workshops commenced, and any changes you observed to them after the workshops?
5. Which aspect(s) of the workshops have had the greatest positive impact on the residents' verbal and nonverbal communication? Why?
6. Can you describe any changes you observed in the social relationships between residents after the workshops?

**Questions for the workshop leader**

1. Can you describe what changes you hoped to see in the residents in the workshops?
2. Can you explain the purpose of using sounds and music in storytelling? How did you decide on the sounds and music to use?
3. Are there particular changes in communication that you hoped for as part of a successful workshop?
4. Can you describe a successful workshop versus an unsuccessful one? What happened with the residents' verbal and nonverbal communication in a successful workshop versus an unsuccessful one?
5. What observable changes do you see or hear in the residents’ verbal and nonverbal communication after the workshops?
6. What changes would you make to the workshops to further improve the residents' verbal and nonverbal communication in the future?
